# Complete Genome Sequence of *w*Bp, the *Wolbachia* Endosymbiont of Brugia pahangi FR3

**DOI:** 10.1128/MRA.00480-20

**Published:** 2020-07-02

**Authors:** Jarrett F. Lebov, John Mattick, Silvia Libro, Benjamin C. Sparklin, Matthew Chung, Robin E. Bromley, Suvarna Nadendla, Xuechu Zhao, Sandra Ott, Lisa Sadzewicz, Luke J. Tallon, Michelle L. Michalski, Jeremy M. Foster, Julie C. Dunning Hotopp

**Affiliations:** a Institute for Genome Sciences, University of Maryland School of Medicine, Baltimore, Maryland, USA; b Division of Protein Expression and Modification, New England Biolabs, Ipswich, Massachusetts, USA; c Department of Biology and Microbiology, University of Wisconsin Oshkosh, Oshkosh, Wisconsin, USA; d Department of Microbiology and Immunology, University of Maryland School of Medicine, Baltimore, Maryland, USA; e Greenebaum Cancer Center, University of Maryland School of Medicine, Baltimore, Maryland, USA; Broad Institute

## Abstract

Lymphatic filariasis is a devastating disease caused by filarial nematode roundworms, which contain obligate *Wolbachia* endosymbionts. Here, we assembled the genome of *w*Bp, the *Wolbachia* endosymbiont of the filarial nematode Brugia pahangi, from Illumina, Pacific Biosciences, and Oxford Nanopore data. The complete, circular genome is 1,072,967 bp.

## ANNOUNCEMENT

Brugia pahangi is a zoonotic filarial nematode and sister taxon to Brugia malayi, a causative agent of human lymphatic filariasis. We sequenced the *Wolbachia* endosymbiont *w*Bp from B. pahangi FR3. B. pahangi FR3, which was originally obtained from a green leaf monkey in Kuala Lumpur (1), is distributed by the NIAID Filariasis Research Reagent Resource Center (FR3) (2).

We assembled the *w*Bp genome using multiple technologies. For short-read sequencing, genomic DNA from B. pahangi FR3 was acquired from BEI Resources, KAPA Hyper libraries were constructed, and 107,643,863 Illumina HiSeq 2500 paired-end 150-bp reads were generated, with quality control and trimming using FastQC v0.11.7 (3) and Trimmomatic v0.38 (4), respectively. Default parameters were used unless otherwise noted. For PacBio RS II sequencing, B. pahangi FR3 genomic DNA was isolated with Qiagen gravity-flow Genomic-tips from 16 male and 16 female worms, which were homogenized in Qiagen G2 buffer containing RNase A with 80 U of proteinase K and precipitated with 20 μg of glycogen. Genomic DNA was fragmented via Covaris gTUBE centrifugation. Libraries were constructed with the SMRTbell v1.0 template preparation kit and size selected (>15 kbp) with a Sage Blue Pippin cassette, and 3,267,281 reads were sequenced with P6C4 chemistry (*N*_50_, 25.4 kbp). For Oxford Nanopore Technologies (ONT) sequencing, 185 adult female worms from TRS Labs (Athens, GA, USA), which maintains worms that are used interchangeably with worms from FR3, were homogenized in liquid nitrogen, and genomic DNA was phenol-chloroform extracted, ethanol precipitated, and spooled. Three ONT SQK-RAD004 libraries were constructed, from 1.8, 0.9, and 0.18 μg genomic DNA, and 553,429 reads, 11,638 reads, and 18,303 reads, respectively (*N*_50_, 21.6 kbp, 39.3 kbp, and 48.2 kbp, respectively), were generated using an R9.4 flow cell (FLO-MIN106), replacing loading beads with water. Additionally, an SQK-RAD004 library was constructed with 4 μg of genomic DNA, 1.5 μl of fragmentation mixture, and 3.5 μl of 10 mM Tris-Cl (pH 8.0) with 0.02% Triton X-100 (5), yielding 143,988 reads (*N*_50_, 19.3 kbp) with an R9.4 flow cell (FLO-MIN106). All ONT reads were base called with Guppy v3.1.5.

A preliminary *w*Bp contig was generated in a *de novo* assembly of all Brugia pahangi data using Canu v1.8 (6), with an estimated genome size of 100 Mbp (7). The *w*Bp contig was identified after comparison of this assembly with the genomes of *w*Bm (the *Wolbachia* endosymbiont of B. malayi [8]) and B. malayi (9) using NUCmer v3.0 (10). Using alignments against this preliminary *w*Bp contig, we extracted 1,100,975 Illumina reads with BWA-MEM v0.7.17 (11) using aM options (153× depth), 6,483 PacBio reads with minimap2 v2.17 (12, 13) using ax map-pb options (*N*_50_, 30.2 kbp; 87× depth), and 11,374 ONT reads with minimap2 v2.17 using ax map-ont options (*N*_50_, 19.9 kbp; 92× depth). These extracted reads were assembled into a circular genome with Unicycler v0.4.8 (14). To resolve indel errors, the entire B. pahangi assembly (7), including the *w*Bp contig, was corrected with Pilon v1.22 (mindepth, 5; K, 85; minmq, 0; minqual, 35; fix indels) (15) using all Illumina reads. The entire B. pahangi assembly was used because of known lateral gene transfers between *w*Bp and B. pahangi, which confound such algorithms (16). The resulting 1,072,967-bp genome (GC content, 34.2%) was manually rotated to the *Wolbachia ori* gene (17), and 1,019 open reading frames, 34 tRNA genes, and 1 copy each of 5S, 16S, and 23S rRNA genes were identified with the IGS Prokaryotic Annotation Pipeline (18).

### Data availability.

This genome has been deposited in GenBank under accession number CP050521. The raw data have been deposited in the SRA under the accession numbers SRX4135331, SRX4135330, SRX4135329, SRX4135328, SRX4135327, SRX4135326, SRX4135325, SRX4135324, SRX4135323, SRX4135322, SRX4135321, SRX4135320, SRX4135319, SRX4135318, SRX4135317, SRX4135316, SRX4135315, SRX4135314, SRX4135313, and SRX4135312 (PacBio), SRX7658407, SRX7658383, SRX7658378, SRX7658377, SRX7658352, SRX7658349, SRX7658341, SRX7658327, SRX7658323, SRX7658322, SRX7658317, and SRR10997235 (Illumina), and SRR11565851, SRR11472020, SRR11565849, and SRR11565826 (MinION).
